# Functional analysis of the 11q23.3 glioma susceptibility locus implicates *PHLDB1* and *DDX6* in glioma susceptibility

**DOI:** 10.1038/srep17367

**Published:** 2015-11-27

**Authors:** Rebekah Baskin, Nicholas T. Woods, Gustavo Mendoza-Fandiño, Peter Forsyth, Kathleen M. Egan, Alvaro N.A. Monteiro

**Affiliations:** 1 Cancer Epidemiology Program, H. Lee Moffitt Cancer Center and Research Institute, Tampa, FL, USA; 2 Eppley Institute for Research in Cancer, University of Nebraska Medical Center, Omaha, NE 68198, USA; 3 Department of Neuro-oncology, H. Lee Moffitt Cancer Center and Research Institute, Tampa, FL, USA

## Abstract

Glioma is the most common malignant primary brain tumor and is associated with poor prognosis. Genetic factors contributing to glioma risk have recently been investigated through genome-wide association studies (GWAS), implicating seven independent glioma risk loci in six chromosomal regions. Here, we performed an in-depth functional analysis of the risk locus proximal to the *PHLDB1* gene on 11q23.3. We retrieved all SNPs in linkage disequilibrium (r^2^ ≥ 0.2) with the glioma-associated SNP (rs498872) and performed a comprehensive bioinformatics and experimental functional analysis for the region. After testing candidate SNPs for allele-specific activity in a luciferase-based enhancer scanning assay, we established a subset of 10 functional SNPs in the promoters of *PHLDB1* and *DDX6,* and in a putative enhancer element. Chromatin conformation capture (3C) identified a physical interaction between the enhancer element containing a functional SNP (rs73001406) and the promoter of the *DDX6* gene. Knockdown experiments in cell culture and 3D assays to evaluate the role of *PHLDB1* and *DDX6* suggest that both genes may contribute to the phenotype. These studies reveal the functional landscape of the 11q23.3 glioma susceptibility locus and identify a network of functional SNPs in regulatory elements and two target genes as a possible mechanism driving glioma risk association.

Gliomas comprise 30% of all primary brain tumors and 80% of malignant brain tumors. Glioma subtypes include oligodendroglioma, oligoastrocytoma, ependymoma and astrocytoma, among other more rare subtypes, as classified by the World Health Organization (WHO)^1^,^2^. Gliomas are categorized based on the cell type of origin and tumor grade, and there is debate concerning the cell type of origin for these complex tumors^2^. GBM is a grade IV astrocytoma that represents more than half of all gliomas and has a very poor prognosis, with a median survival of 12
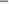
14 months with optimal therapy^2^. Low-grade gliomas (LGG) are typically associated with longer survival, but still have poor outcomes and can recur or advance to grade III or IV tumors^2^.

There are relatively few risk factors known to contribute to glioma development, either environmental or genetic. Because of a need for better risk assessment and therapeutic strategies, several groups in recent years have conducted genome-wide association studies (GWAS) in order to identify single nucleotide polymorphisms (SNPs) that are associated with glioma susceptibility. These studies identified seven independent glioma risk loci in six chromosomal regions: 5p13.33 (*TERT*), 7p11.2 (*EGFR*, two loci), 8q24.21 (*CCDC26*), 9p21.3 (*CDKN2A/B*), 11q23.3 (*PHLDB1*) and 20q13.33 (*RTEL1*)^3^,^4^,^5^,^6^,^7^,^8^,^9^. Since the discovery of these glioma risk-associated loci, progress to functionally characterize them has been relatively slow. Many of the loci identified are pleiotropic, and several of them contain well-known cancer-related genes (*TERT, EGFR*, and *CDKN2A/B*). However, thorough functional analysis of each locus is necessary to confirm a causal relationship to susceptibility.

Functional analysis of cancer susceptibility loci is a field that has developed rapidly over the last few years. GWAS typically uncover SNPs that do not alter protein structure or function, but rather lie in non-coding regions and are therefore more difficult to characterize. A large portion of these SNPs in non-coding regions are believed to act by modulating the activity of regulatory regions in which they reside, but can also influence splicing, repression, micro-RNA (miRNA) function, or may work by other unknown mechanisms^10^,^11^. Recently, the availability of large-scale data from the Encyclopedia of DNA Elements (ENCODE) project and the development of novel experimental methods have improved upon our ability to characterize these non-coding SNPs^10^,^11^,^12^,^13^. Here, we have applied a systematic functional analysis to identify candidate functional SNPs and target genes within the 11q23.3 glioma susceptibility locus, a locus with relatively little annotation related to cancer predisposition. We began with a bioinformatics analysis of all SNPs in linkage disequilibrium (LD) (r^2^ ≥ 0.2) with the *PHLDB1* tag SNP, rs498872, which led to a total of 41 candidate functional SNPs. We also conducted an analysis of genes within the locus in order to identify potential target genes. Experiments using normal human astrocyte (NHA) and human malignant glioma (U87MG) cells were conducted in order to assess the enhancer activity of each SNP and potential influence on protein binding or chromatin interactions. Finally, a 3D culture model system (neurospheres) was used to assess two potential target genes within the locus for their functional relevance.

## Results

### Identification of candidate SNPs

**Figure 1:**
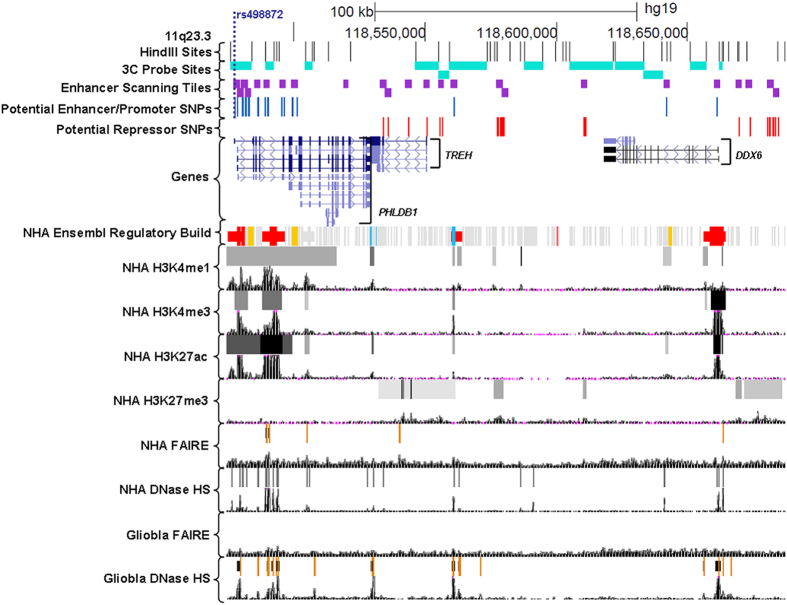
Regulatory landscape and experimental approach at the 11q23.3 glioma susceptibility locus. Tracks are denoted on the left panel. *Hind*III restriction sites were used to design 3C probes (turquoise). Enhancer scanning tiles (purple) were designed to cover regulatory elements containing potential enhancer SNPs (blue) or repressor SNPs (red). Genes were obtained from ENCODE RefSeq. All remaining tracks were obtained from ENCODE publicly available data.

In order to identify candidate functional SNPs we retrieved all (n = 96) SNPs in LD with the associated SNP (rs498872) at a threshold of r^2^ ≥ 0.2 (Supplementary Table 1). The GWAS-identified SNP, rs498872, lies within the 5′-UTR of the *PHLDB1* gene. All 96 SNPs lie within an approximate 600 kb region spanning *PHLDB1, TREH* and *DDX6* genes (Supplementary Fig. 1). Application of a tissue-specific bioinformatics pipeline revealed 41 candidate functional SNPs distributed over a smaller 200 kb region (Fig. 1), 15 of which lie within enhancer or promoter regions characterized by the presence of one of the following marks, also called biofeatures: Histone H3 Lysine 4 mono-methylation (H3K4me1, marker of enhancers and promoters), Histone H3 Lysine 4 tri-methylation (H3K4me3, marker of promoters), or Histone H3 Lysine 27 acetylation (H3K27Ac, marker of active enhancers) in NHA cells, or FAIRE-seq or DHS peaks (DNAse I hypersensitive sites; markers of open chromatin) in NHA or Gliobla cells^14^ (Fig. 1) (see methods for rationale for choice of cell lines). The remaining 26 SNPs lie within potential repressors, as indicated by Histone H3 Lysine 27 tri-methylation (H3K27me3) in NHA cells (Fig. 1).

### Identification of candidate target genes

Next, using the 600 kb region defined by 96 candidate SNPs we identified the boundary SNPs and added 1 Mb to each end of the locus, delimiting a region of 2.6 Mb (Supplementary Fig. 1). This region includes 61 candidate target genes. This distance was chosen based on the observation that interactions between enhancers and promoters are not limited to a haplotype block but the majority of enhancers act on genes within 1 Mb of their chromosomal coordinates^15^. Thus, every gene contained in this 2.6 Mb region is considered a candidate target. We conducted an *in silico* analysis of the genes within this region using publicly available data. Interestingly, we found a consistently higher rate of alterations of these genes in LGG when compared to GBM according to The Cancer Genome Atlas (TCGA) records from cBioPortal (Supplementary Fig. 2)^16^,^17^. This observation is consistent with glioma GWAS findings, which report that the rs498872 variant is associated with LGG but not with GBM. This pattern was also observed in genes in the 8q24.21 (*CCDC26*) locus previously associated with LGG but not in the 9p21.3 (*CDKN2A/B*) locus previously associated with high grade glioma, where the pattern is reversed (Supplementary Fig. 2)^3^,^18^. We also conducted searches in COSMIC for information on somatic mutations in all genes within the locus, either in any cancer or specifically in brain cancers. While most of the genes in the locus display somatic alterations in any cancer, only a small subset are mutated in brain tumors, notably *PHLDB1* and *DDX6* (Supplementary Fig. 3). In order to assess expression of these genes in normal brain or glioma tissue, we examined H3K4me3 and RNA-seq data in ENCODE. Several genes within the locus had no expression in either tissue (Supplementary Fig. 3).

### Enhancer scanning and EMSA

**Figure 2:**
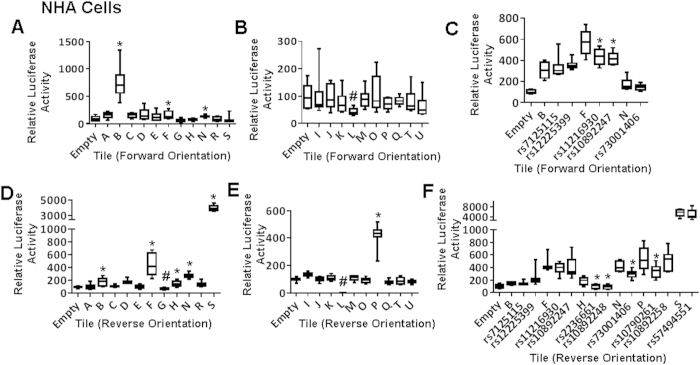
Enhancer scanning at the 11q23.3 locus in NHA cells. NHA cells were transfected with luciferase constructs for enhancer elements (**A,D**) or repressor elements (**B,E**). Tiles with enhancer activity were evaluated for allele-specific activity (**C,F**), with SNP minor alleles to the right of their corresponding reference tile. (**A,B,D,E**) ^*^significant enhancer activity; ^#^significant repressor activity. (**C,F**) ^*^significant difference between major and minor allele. (p < 0.05).

**Figure 3:**
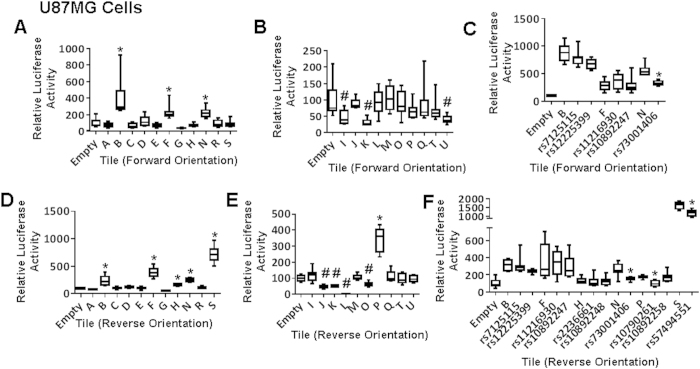
Enhancer scanning at the 11q23.3 locus in U87MG cells. U87MG cells were transfected with luciferase constructs for enhancer elements (**A,D**) or repressor elements (**B,E**). Tiles with enhancer activity were evaluated for allele-specific activity (**C,F**), with SNP minor alleles to the right of their corresponding reference tile. (**A,B,D,E**) ^*^significant enhancer activity; ^#^significant repressor activity. (**C,F**) ^*^significant difference between major and minor allele. (p < 0.05)

We conducted enhancer scanning assays^19^,^20^ on each region containing the 41 candidate SNPs from our bioinformatics analysis. Tiles of approximately 2 Kb were designed to cover the biofeatures overlapping with candidate SNPs (Fig. 1). These tiles were cloned into a luciferase reporter vector and transfected into U87MG and NHA cells. Tiles showing activity in at least one orientation in either cell line were explored further. Enhancer scanning implicated ten SNPs in five tiles with enhancer activity, but only seven of these SNPs demonstrated allele-specific luciferase activity in NHA and/or U87MG cells (Figs 2 and 3). Two of the seven SNPs lie within a region near repressor marks rather than enhancer marks. Nine SNPs within six tiles showed significant repressor activity (Figs 2 and 3). Interestingly, two of these nine lie within a tile overlapping ENCODE enhancer marks, but this region consistently showed repressor activity in our assay.

**Figure 4:**
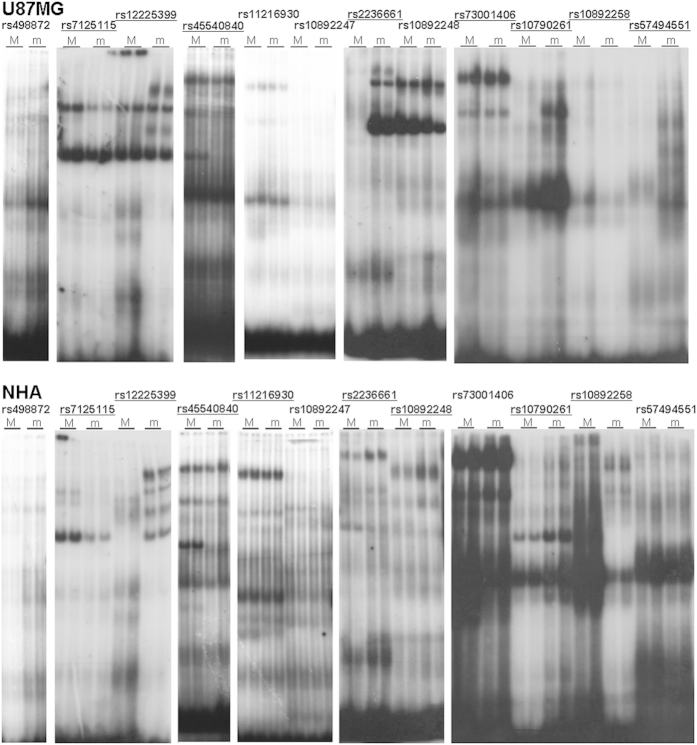
Protein binding of 11q23.3 enhancer SNPs by EMSA. EMSAs were conducted with probes containing major and minor alleles for all positive enhancer SNPs using lysate from U87MG (top) and NHA (bottom). Underlining indicates SNPs with allele specific binding. M = major allele, m = minor allele.

**Figure 5:**
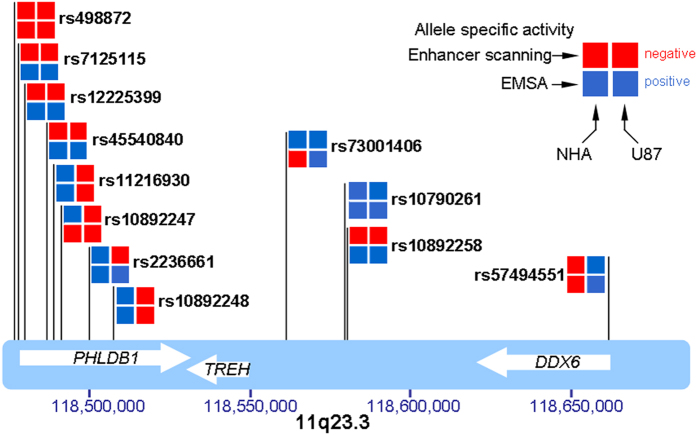
Summary of enhancer scanning and EMSA for 11q23.3 enhancer SNPs. SNPs are shown in relation to their genomic coordinates. Results are represented as positive (+, blue) or negative (–, red) for allele specificity in each assay and each cell line.

In order to determine if the presence of the risk allele had an impact on the protein binding capability of each DNA fragment, we conducted EMSA for all regions that showed enhancer activity in the luciferase assays. We tested ~40 bp fragments containing the major or minor allele for each of the 10 SNPs within active tiles. To be comprehensive, we also chose to test the GWAS-tagged SNP (rs498872) and rs45540840 because of its relatively high LD (r^2^ ≥ 0.5) with the tagged SNP, for a total of 12 SNPs. EMSA showed allele-specific protein binding in U87MG and/or NHA cells for nine of the ten SNPs, as well as for the one additional high LD SNP rs45540840 (Fig. 4). No allele-specific binding was observed for the tag SNP, rs498872. Eleven candidate functional SNPs were reproducibly positive in the enhancer scanning or EMSA, in at least one cell line (Table 1) (Fig. 5). Next, we used MATCH™ (http://www.gene-regulation.com/pub/programs.html#match) to identify transcription factors with differential binding to major and minor alleles, which revealed that the majority of these SNPs are predicted to affect binding of at least one factor (Supplementary Table 2). This suggests that several functional SNPs are likely to contribute to regulation in the locus.

### Chromosome conformation capture (3C)

After conducting enhancer scanning to identify candidate functional SNPs, we examined the positive hits for their locations relative to any gene promoters in the region. Of the SNPs within active tiles, six lie on or near the promoter regions of two *PHLDB1* transcripts and one lies on the promoter of *DDX6* (Table 1). Thus, these SNPs implicate *PHLDB1* and *DDX6* as candidate target genes in the locus.

**Figure 6:**
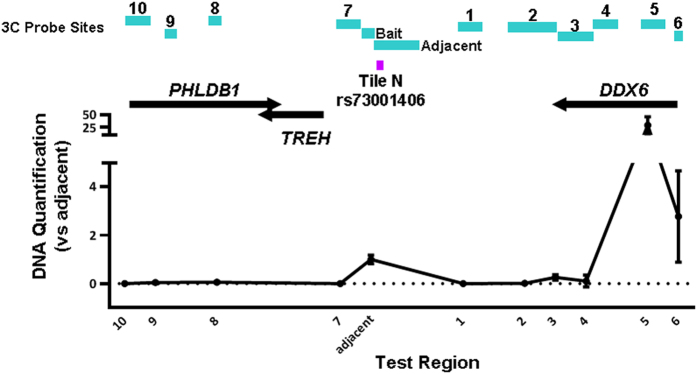
3C at the 11q23.3 locus. 3C was conducted in U87MG cell lines to examine interactions between Tile N, containing rs73001406, and promoters within the region.

The remaining three SNPs lie between *TREH* and *DDX6* and are at least 10 kb from any transcription start site (Fig. 5). Two of these lie within a tile that overlaps with repressive H3K27me3 marks in NHA cells, but overlaps with no other functional elements in NHA or U87MG cells. SNP rs73001406 lies within a tile that overlaps with a very sharp peak for H3K27Ac, H3K4me3, H3K4me1, DHS and FAIRE-seq in NHA cells as well as a sharp FAIRE-seq peak in Gliobla cells (Fig. 1). This particular SNP also demonstrated allele-specific luciferase activity, with the minor allele showing significantly reduced activity when compared with the major allele. We examined publicly available RNA Polymerase II ChIA-PET data from 4 cell lines in ENCODE^21^ and found a very strong interaction between the region containing rs73001406 and the promoter region of *DDX6*, which is approximately 100 kb away, albeit in non-brain related K562 and MCF7 cells (Supplementary Fig. 4). Therefore, this SNP was considered a candidate for conducting 3C in order to determine if its enhancer region interacts with promoter elements of any surrounding genes in glioma-relevant cells. We used *Hind*III restriction sites in order to design 3C probes spanning the region from *PHLDB1* to *DDX6*, with the tile containing rs73001406 as the anchor region. 3C analysis using U87MG cells showed a strong interaction between the anchor region and the promoter of *DDX6* (Fig. 6, Supplementary Table 6). No significant interaction was detected between the anchor and the proximal core promoters of either *TREH* or *PHLDB1*, both of which lie centromeric to the regulatory element. Therefore, we conclude that this element interacts specifically with the *DDX6* promoter in glioma cells.

### Neurosphere Formation

**Figure 7:**
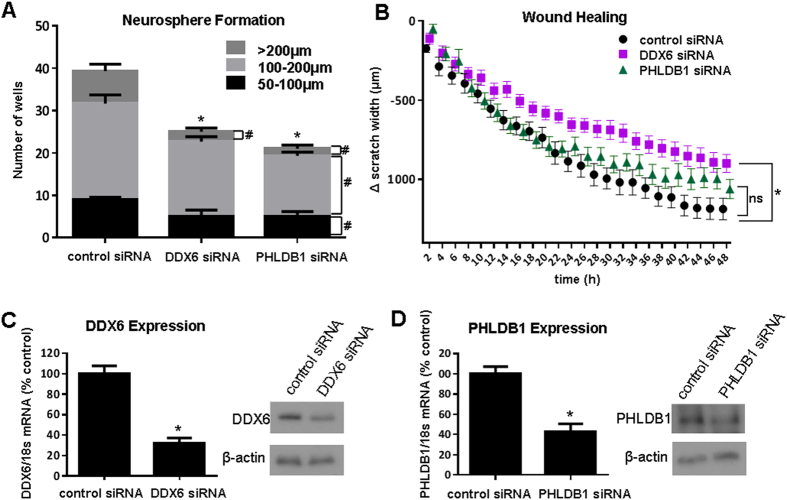
Effects of DDX6 and PHLDB1 knockdown on U87MG neurosphere formation and migration. Neurosphere formation (**A**) and cell migration (**B**) were quantified in U87MG cells transfected with a negative control siRNA, DDX6 siRNA, or PHLDB1 siRNA. Knockdown was confirmed by qRT-PCR and Western blot (**C,D**). (**A**) ^*^significant reduction in total number of neurospheres (p < 0.05); ^#^significant reduction in neurospheres in each size category (p < 0.05). (**B**) ^*^p < 0.05 vs control siRNA. (**C,D**) ^*^p < 0.05 vs control siRNA.

The neurosphere formation assay is a technique that allows assessment of the self-renewal and differentiation capacity of neural stem cells^22^,^23^. It has been adapted for the purpose of evaluating glioma cells and is often used to assess their stem-like properties and tumorigenic potential^24^. It has been demonstrated that U87MG cells are capable of readily forming neurospheres in culture^25^,^26^. Therefore, we used this cell line as a model to test the effects of target gene expression within the 11q23.3 locus on neurosphere formation. The assay was performed using U87MG cells transfected with siRNAs targeting *DDX6* or *PHLDB1*. Knockdown efficiency ranged from 60 to 70% according to quantitative RT-PCR (Fig. 7C,D). Cells were then plated at a concentration of 0.5 cells per well in a 96-well plate in neurosphere induction media. After 14 days, the number and size of neurospheres per well were quantified for each group. We found that knockdown of *DDX6* and *PHLDB1* led to a reduction in the total number of neurospheres, as well as a reduction in the percent of neurospheres with a size greater than 200 μm (Fig. 7A).

In order to determine if impairments in cell proliferation or induction of cytotoxicity may play a role in the effects of gene knockdown on neurosphere formation, we conducted MTS (3-(4,5-dimethylthiazol-2-yl)-5-(3-carboxymethoxyphenyl)-2-(4-sulfophenyl)-2H-tetrazolium, inner salt), LDH (Lactate Dehydrogenase) release, and cell count assays. U87MG cells were transfected with each siRNA and were plated in 96-well plates for each assay. We found that at 72h, proliferation as measured by MTS assay was significantly lower with knockdown of *DDX6* or *PHLDB1* than with negative control siRNA (Supplementary Fig. 5A). However, we found that only knockdown of *PHLDB1* caused a significant increase in LDH release or a significant decrease in cell number (Supplementary Fig. 5B,C). These results indicate that the effect of *PHLDB1* gene knockdown on neurosphere formation can be attributed to increased cell death. However, *DDX6* knockdown effects on reduced viability and neurosphere formation cannot be explained by reduced proliferation or increased cell death.

### Wound Healing

Cell migration is an important property of glioma cells and contributes to their ability to invade surrounding brain tissue^26^. Therefore, we assessed the effects of knocking down the two candidate target genes, *PHLDB1* or *DDX6*, in U87MG cells (Fig. 7C,D). Cells were then plated in a 96-well plate and wound healing assays were performed. The change in scratch width over time was used to assess cell migration. We found that knockdown of *DDX6* led to a significant reduction in the migratory phenotype of U87MG cells, while knockdown of *PHLDB1* had no significant effect (Fig. 7B). These results indicate that *DDX6* expression may influence glioma cell migration.

## Discussion

Here, we have conducted a functional analysis of the 11q23.3 glioma risk locus in order to explore potential mechanisms of susceptibility. Our overall goal was to identify regulatory SNPs within the locus and their target genes, and to understand how these genes may contribute to glioma risk. We initially identified 41 candidate SNPs, 10 of which lie in regions with enhancer activity in NHA or U87MG cells. The majority of these SNPs lie within promoter regions, either for *PHLDB1* or *DDX6*, while the remaining SNPs lie in additional enhancer or repressor regions. 3C experiments detected a physical interaction between one enhancer region containing a candidate SNP and the promoter of *DDX6*. Knockdown of *PHLDB1* in U87MG cells had a significant impact on cell viability due to increased cell death. Knockdown of *DDX6* also had a significant impact on cell viability, cell migration, and neurosphere formation that cannot be explained by increased cell death or reduced proliferation. Although we do not know the mechanisms by which *DDX6* exerts these effects, it is plausible that it affects cell processes involved in self-renewal. Interestingly, a recent report has demonstrated that silencing of *DDX6* results in premature differentiation of human epidermal tissue^27^. In this model, DDX6 promotes the degradation of differentiation-inducing transcripts or the translation of self-renewal/proliferation mRNAs^27^. Taken together, these data combined with the *in silico* analysis, indicate that *PHLDB1* and *DDX6* constitute the likely target genes regulated by our candidate SNPs.

DEAD box helicase 6 (*DDX6*) is an RNA helicase and a member of the DEAD (Asp-Glu-Ala-Asp) box protein family. *DDX6* plays an important role in translational repression by binding to mRNA and sequestering it to P-bodies, followed by recruitment of the decapping complex^28^. *DDX6* is essential in miRNA-mediated gene silencing^29^ and down regulates miR-143/145^30^. The 11q23.3 locus also contains the *MLL* gene and is amplified in acute myeloid leukemia (AML) and myleodysplastic syndrome (MDS). *DDX6* was identified as the second gene within this region, after *MLL*, to be specifically up regulated in myeloid malignancies even in the absence of 11q23.3 amplification^31^. Thus *DDX6* may play a role in multiple cancers, and our results implicate *DDX6* in the potential mechanisms contributing to glioma risk.

The other top candidate gene in the locus, pleckstrin homology-like domain family B member 1 (*PHLDB1*), is a relatively poorly characterized gene. PHLDB1, also known as LL5α, contains a PH domain, a Forkhead-associated (FA) domain and a structural maintenance of chromosomes ATPase (SMC) domain^32^. It facilitates insulin-dependent Akt phosphorylation and GLUT4 translocation in adipocytes, potentially through membrane localization via its PH domain^33^. It has also been shown to play a role in laminin-dependent microtubule anchoring at the epithelial cell basal cortex^34^. Our results provide evidence for a new functional role of this gene in glioma.

A glioma fine-mapping study in the Han Chinese population revealed rs17748, rs2236661, and rs494560 as potential correlated SNPs in the 11q23.3 locus^35^. Two of these (rs17748 and rs2236661) are in LD (r^2^ = 0.53) with rs498872. One SNP, rs2236661, lies within an enhancer region that showed activity in our luciferase assays, while rs17748 and rs494560 do not overlap with any functional elements in brain cells. Therefore, these two SNPs were not further investigated in our functional dataset.

Our data strongly implicate rs73001406 as an important functional SNP within this locus. It is notable that the region containing rs73001406 overlaps both promoter and enhancer marks (Supplementary Table 1), which suggests that the region could act as a distal promoter for the *TREH* gene. However, this gene is not expressed in normal brain or glioma cell lines according to ENCODE data (Supplementary Fig. 3) and the Human Protein Atlas^36^, and the regulatory element lies 10kb from the transcription start site. Our results and the previous ChIA-PET findings strongly link the rs73001406 region to *DDX6* regulation, but a potential impact on *TREH* expression cannot be ruled out.

Due to the absence of dense fine-mapping in the locus we cannot rule out the possibility that rare variants may also contribute the regulation of the locus. In addition, we identified a number of SNPs in this study that may have repressor activity in astrocyte and/or glioma cells. The functional consequences of these SNPs remain unknown and will be the subject of future work. Despite these limitations, we provide a comprehensive examination of the regulatory landscape of the 11q23.3 glioma susceptibility locus, and identify candidate regulatory regions and target genes that are likely to contribute to risk. Further studies will be necessary to fully elucidate the mechanism(s) of glioma association in the locus as well as the respective relative contributions of different elements.

## Materials and Methods

### Retrieval of candidate SNPs and in silico analysis

The glioma-associated SNP from GWAS at the 11q23.3 glioma locus, rs498872, was used to retrieve candidate functional SNPs. Given the lack of fine-mapping data for this region at the initiation of the study, the Broad Institute SNAP Proxy Search tool (http://www.broadinstitute.org/mpg/snap/) was used to retrieve SNPs in LD with the tagged SNP at a relatively low LD threshold of r^2^ > 0.2.

The resulting 96 SNPs were then subjected to *in silico* functional predictions and annotations using SNPnexus^37^, SNPinfo^38^, Polyphen 2^39^, the UCSC Genome Browser^40^, and RegulomeDB^41^. The results from these searches were organized into a MySQL relational database. The complete RegulomeDB^41^ dataset was also incorporated into this MySQL database. A Python program (SNPFunc_Retriever.py) was used to extract selected data from each of the databases with the associated SNP information. To restrict for cell-type specific functional elements, a manual inspection of publicly available data was conducted using ENCODE for NHA and Gliobla histone modification marks, FAIRE seq, and DHS within the region. See below information for the rationale of choice of cell line data.

### Identification and in silico analysis of candidate target genes

For comprehensive gene analysis, 1 Mb was added to each end of the flanking (outermost) candidate SNPs and all genes within this approximate 2.6 Mb region were considered potential target genes. This list was used to search cBioPortal^16^,^17^ and COSMIC^13^ for potential functional relevance of these genes in all cancers and/or specifically in brain cancers.

### Cell Lines and Cell line Data

As topological and functional determinants operating in each locus might be cell type specific, we paid particular attention to identify cell lines and cell line data relevant for our model. Ideally, we would use normal cells from the tissue in which the tumor originates, but this is not always an available option. Thus, we chose to use, whenever possible, cell lines and datasets relevant to gliomas. Experiments were conducted in *hTERT* immortalized normal human astrocytes (NHA) cells (Applied Biological Materials, Inc.) and/or U87MG glioma cells (ATCC). Cells were cultured in DMEM supplemented with 10% FBS, non-essential amino acids, penicillin-streptomycin, and amphotericin B.

We searched for available regulatory data in ENCODE that would be relevant for the tumor and normal tissue type of interest. Our searches revealed that relevant datasets were available for NHA, Gliobla, M059J, and U87 cells. The NHA cell line had the most comprehensive available regulatory data including FAIRE-Seq, DHS, histone methylation and acetylation tracks. For this reason, we chose NHA as our normal brain cell type for the analysis. M059J and Gliobla had DHS data, but the latter also had available FAIRE-Seq data so we used this cell line as our glioma representative. We also used U87MG RNA-Seq data.

### Enhancer Scanning

Enhancer scanning was performed as previously described^19^. Tiles containing ~2 kb segments of genomic DNA were constructed from bacterial artificial chromosomes (BACs). To cover the 11q23.3 locus we used BACs RP11-45N4 and CTD-2333F20 (Life Technologies). We PCR amplified genomic segments from each BAC using primers containing *att B* recombination sites and a restriction site (Supplementary Table 3). BAC DNA was extracted using a MaxiPrep kit (Qiagen). PCR amplification was carried out using Hotstart Taq polymerase (Qiagen) and PCR products were gel purified. A BP reaction was carried out using Gateway BP Clonase II (Life Technologies) with 50 ng of PCR product and 150 ng pDONR 221 according to manufacturer instructions. BP reactions were used to transform Top10 chemically competent *E. coli*. Colonies were selected and screened by restriction digest. Correct plasmids were then used to carry out LR reactions with Gateway LR Clonase II (Life Technologies) in order to transfer the genomic fragment into the pGL3-LR vector, which we previously constructed using the Gateway Vector Conversion System (Life Technologies). Fragments were cloned into the vector in forward and reverse orientations and tested for luciferase activity.

For enhancer scanning assays, U87MG and NHA cells were used. Cells were plated at 5,000 cells per well in 96-well plates one day prior to transfection. Tiles in the pGL3-LR vector were co-transfected with a pRL-CMV renilla internal control plasmid using Fugene6 (Promega) at a ratio of 3:1 Fugene6 volume (μL) to DNA (μg). After 24 hours, luciferase activity was measured using the Dual Glo Luciferase Assay Kit from Promega. Luciferase values were normalized to the internal control and compared with the empty pGL3-LR control vector. Each tile was assayed in eight technical replicates in two independent experiments for each tile orientation.

### Electrophoretic Mobility Shift Assay (EMSA)

Nuclear proteins from U87MG and NHA cells were extracted using a hypotonic lysis buffer (10 mM HEPES, pH 7.9, 1.5 mM MgCl_2_, 10 mM KCL) supplemented with DTT and protease inhibitors, followed by an extraction buffer (20 mM HEPES, ph 7.9, 1.5 mM MgCL_2_, 0.42 M NaCl, 0.2 mM EDTA, 25% v/v glycerol) supplemented with DTT and protease inhibitors. EMSA probes were designed to cover each SNP plus or minus 20 base pairs, for both major and minor alleles (Supplementary Table 4). Probe pairs were dissolved in TE buffer and annealed at a concentration of 10 μM each. Probes were labeled with ATP [γ-32P] (Perkin Elmer) using T4 polynucleotide kinase (NEB) and cleaned using the QiaQuick Nucleotide Removal Kit (Qiagen). Labeled probes were then incubated with protein extracts using LightShift Poly(dI-dC) (Thermo) and a binding buffer (10 mM Tris, 50 mM KCl, 1 mM DTT, pH 7.4) and electrophoresed on a 6% acrylamide gel overnight at 83 V. Gels were dried and films were exposed for 4–24 h. EMSAs were performed in two technical replicates.

### Chromosome Conformation Capture (3C)

U87MG cells were cultured in 15 cm plates to 80% confluence and were fixed with 1% formaldehyde. The nuclear fraction was isolated using a lysis buffer containing 10mM Tris HCl pH 8, 10 mM NaCl, 0.2% NP40 and a protease inhibitor cocktail (Roche). DNA samples were digested with 375 units of HindIII (NEB) and ligated using T4 DNA ligase (NEB). Samples were then digested with Proteinase K (Qiagen), phenol chloroform extracted and ethanol precipitated prior to PCR analysis. Unligated and ligated samples were analyzed on an agarose gel and a control PCR was performed to confirm successful ligation using control primers described previously^42^. Test primers for each region were designed using Primer3 and were obtained from Sigma or IDT (Supplementary Table 5). Quantitative PCR was performed to analyze fragments within the 11q23.3 locus using Hotstart Taq polymerase (Qiagen). An artificial 3C library was constructed using BAC DNA (RP11-45N4, Invitrogen) and this library was used to confirm primer efficiency and as the standard curve for each 3C assay. 3C assays were performed in three technical replicates in two independent experiments.

### Neurosphere Formation Assay

U87MG cells were transfected with each siRNA (Silencer Select Negative Control 1, *DDX6*, and *PHLDB1* siRNAs from Life Technologies) using Lipofectamine 2000 (Life Technologies) at a final concentration of 10 nM. Media was changed at 6 hours and after 24 hours cells were trypsinized and harvested. To maximize the number of wells with single cells for the neurosphere formation we used a dilution of 5 cells per mL in DMEM/F12 (Life Technologies) supplemented with B27 Supplement (Life Technologies) and growth factors (10 ng/ml bFGF and 20 ng/ml EGF – Life Technologies). Cells were plated in a volume of 100 μL/well in 96-well plates and cultured for 14 days. The number of neurospheres per well was counted and the size of each sphere was measured at the end of the incubation period. Neurosphere assays were performed in three technical replicates in two independent experiments.

### Wound Healing Assay

U87MG or NHA cells were transfected with siRNAs as described for neurosphere formation assays. 24 hours after transfection, cells were counted and seeded at 10K cells per well in a 96-well plate. The next day a scratch was made using a WoundMaker (Essen Biosciences) and washed with media twice before replacing media with DMEM containing 1% FBS. Images were taken every 2 hours for 48 hours in an IncuCyte ZOOM and the change in scratch width was measured. Wound healing assays were performed with eight technical replicates in two independent experiments.

### Cell Viability, Proliferation and Cytotoxicity Assays

U87MG cells were seeded at 2.5 × 10^3^ cells per well in a 96-well plate 24 h after transfection with siRNA. Cell proliferation was measured at various timepoints using the CellTiter 96 Aqueous One Solution Assay (MTS) (Promega) following manufacturer’s instructions. LDH release was measured at various timepoints using the Pierce LDH Cytotoxicity Assay Kit (Life Technologies) following manufacturer’s instructions. Cell viability was measured by counting cells at each timepoint via Trypan blue exclusion. Each assay was performed in three technical replicates and repeated in two independent experiments.

### Quantitative RT-PCR

RNA was extracted using a Qiagen RNeasy mini prep kit. RT was performed using the Qiagen QuantiTect Reverse Transcription Kit with genomic DNA removal. Quantitative PCR was performed using TaqMan gene expression assays for DDX6, PHLDB1 and 18 s with TaqMan Universal PCR Master Mix (Life Technologies).

### Western Blotting

Protein was extracted using NETN lysis buffer and equal amounts of protein were run on a 10% gel. Proteins were transferred to PVDF membranes and blotted with the following primary antibodies: DDX6 (Abcam, rabbit, 1:1000), PHLDB1 (Abcam, rabbit, 1:1000), and β-actin (Sigma, mouse, 1:2000). HRP-conjugated secondary antibodies were used.

### Statistical Analysis

Enhancer scanning, neurosphere formation and quantitative RT-PCR results were analyzed using Student’s t-test. Wound healing assay, cell proliferation, cytotoxicity, and cell viability assays were analyzed by two-way ANOVA.

## Additional Information

**How to cite this article**: Baskin, R. *et al.* Functional analysis of the 11q23.3 glioma susceptibility locus implicates *PHLDB1* and *DDX6* in glioma susceptibility. *Sci. Rep.* **5**, 17367; doi: 10.1038/srep17367 (2015).

## Supplementary Material

Supplementary Information

## Figures and Tables

**Table 1:** Top Candidate SNPs from Enhancer Scanning and EMSA Results.

RSID	hg19 coordinates	Tile Location	LD to rs498872	Overlapping Regulatory Elements
rs7125115	chr11:118478330	B	0.268	PHLDB1 promoter
rs12225399	chr11:118480285	B	0.241	PHLDB1 promoter
rs45540840	chr11:118486110	E	0.507	PHLDB1 promoter/enhancer
rs11216930	chr11:118488782	F	0.507	PHLDB1 promoter
rs10892247	chr11:118490076	F	0.507	PHLDB1 promoter
rs2236661	chr11:118499394	H	0.477	PHLDB1 promoter/enhancer
rs10892248	chr11:118501022	H	0.477	PHLDB1 promoter/enhancer
rs73001406	chr11:118560857	N	0.383	Enhancer/CTCF binding
rs10790261	chr11:118579747	P	0.266	H3K27me3 (repression)
rs10892258	chr11:118579865	P	0.266	H3K27me3 (repression)
rs57494551	chr11:118661398	S	0.208	DDX6 promoter
