# Supplementary material for: Functional analysis of the 11q23.3 glioma susceptibility locus implicates PHLDB1 and DDX6 in glioma susceptibility
Source: Sci Rep. 2015 Nov 27;5:17367. doi: 10.1038/srep17367 (PMC4661592; doi:10.1038/srep17367)
Supplement: Supplementary Information [file srep17367-s1.pdf]

Supplementary Material

Functional analysis of the 11q23.3 glioma susceptibility locus implicates *PHLDB1* and *DDX6* in glioma susceptibility

Rebekah Baskin[1], Nicholas T. Woods[1,2], Gustavo Mendoza-Fandiño[1], Peter Forsyth[3], Kathleen M. Egan[1], Alvaro N.A. Monteiro[1]

[1]Cancer Epidemiology Program, H. Lee Moffitt Cancer Center and Research Institute, Tampa, FL, USA

[2]Eppley Institute for Research in Cancer, University of Nebraska Medical Center, Omaha, NE 68198, USA

[3]Department of Neuro-oncology, H. Lee Moffitt Cancer Center and Research Institute, Tampa, FL, USA

Correspondence should be addressed to:
Alvaro N. Monteiro
H. Lee Moffitt Cancer Center & Research Institute
12902 Magnolia Drive, Tampa, FL 33612, USA.
Phone: 813-7456321Fax: 813-9036847
E-mail: alvaro.monteiro@moffitt.org

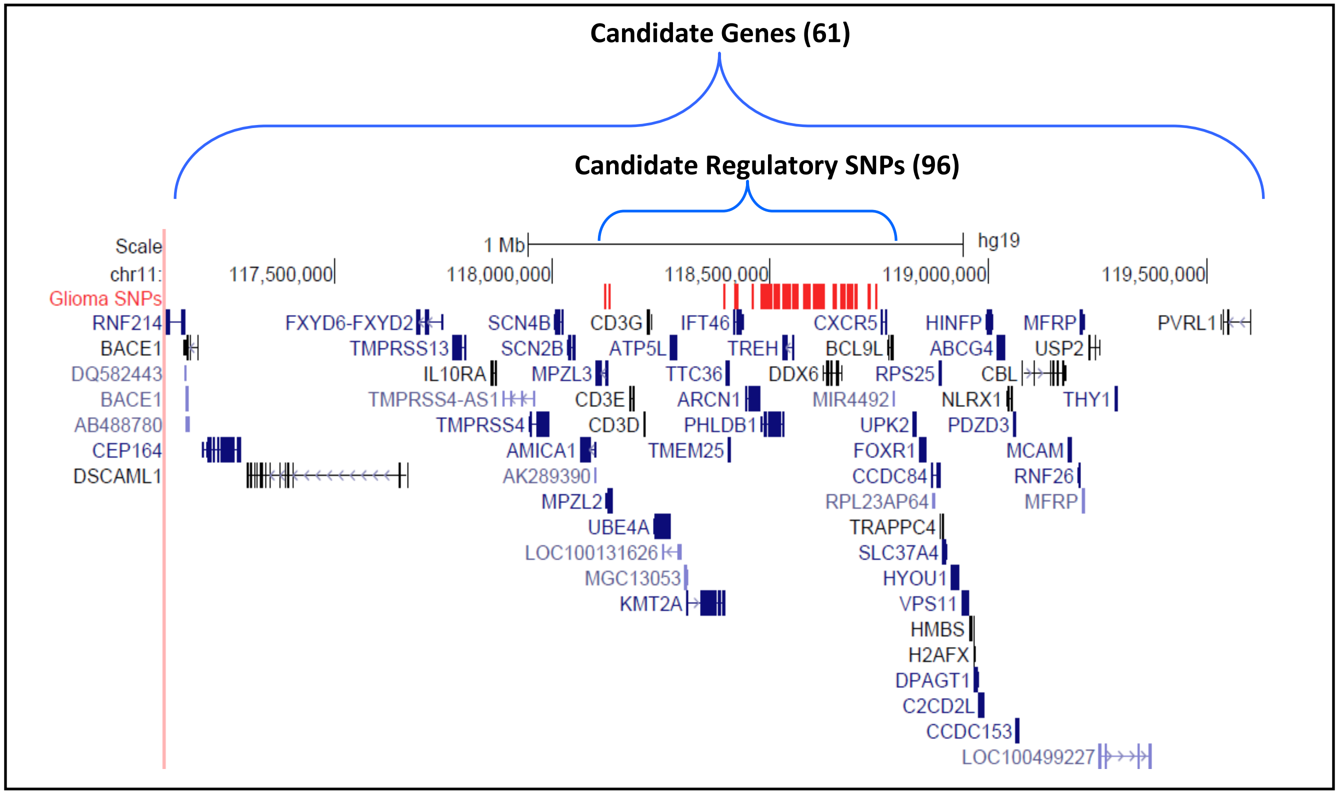

Supplementary Figure 1: All genes and candidate SNPs within the 11q23.3 glioma susceptibility locus. Candidate regulatory SNPs are shown as light blue tick marks. A 1Mb boundary was established on either end of the outermost SNPs. The total 2.6Mb region includes 61 candidate genes.

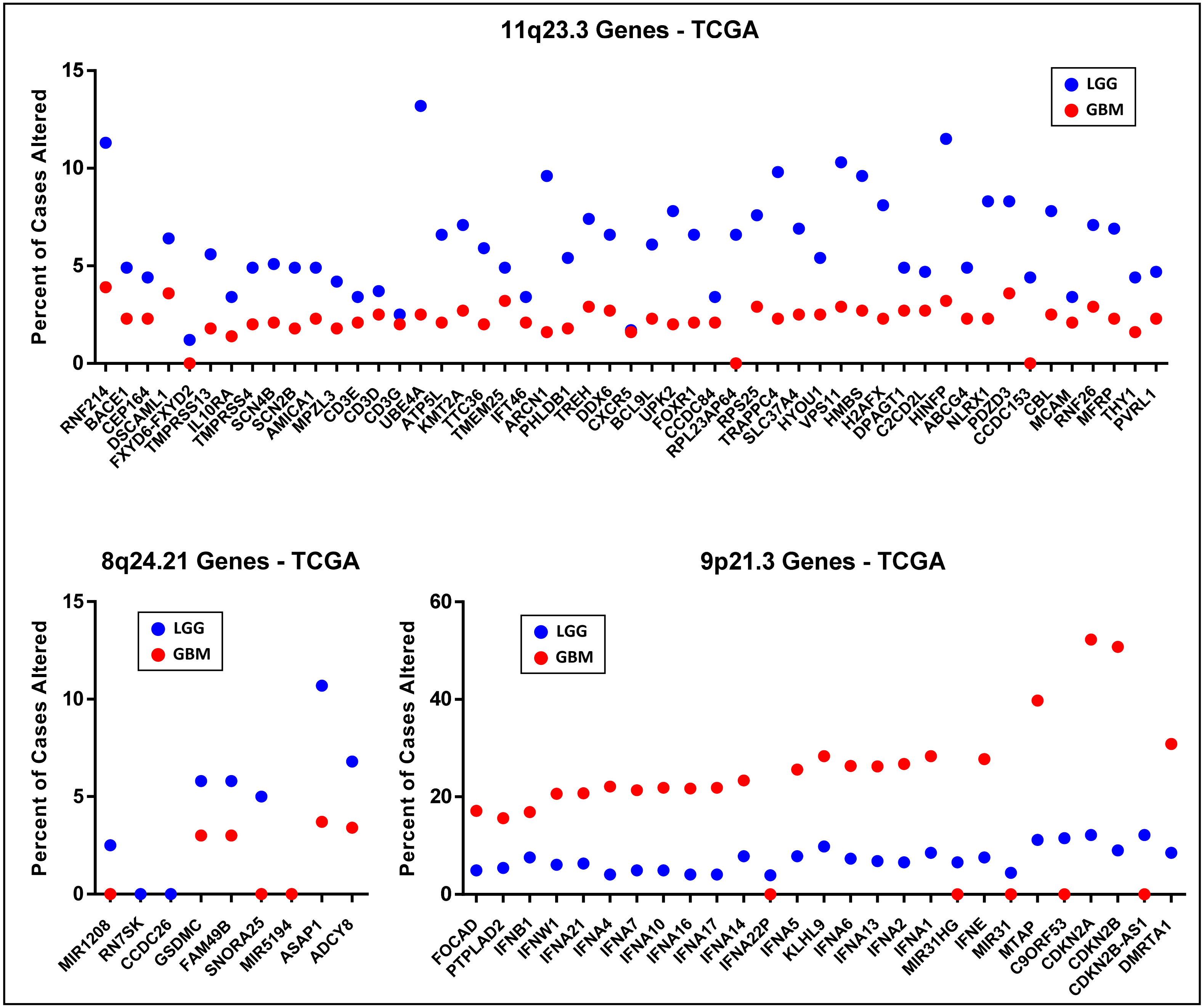

Supplementary Figure 2: Role of 11q23.3 locus genes in cancer. Gene lists were entered into TCGA cBioPortal database searches to obtain mutation, CNV and expression data. The frequency of alterations in each gene were compared between LGG and GBM cases for 11q23.3, 8q24.21 and 9p21.3 glioma loci.

	COSMIC mutations							ENCODE	
	missense		insertion		deletion			Promoter marks in NHA	RNA-seq in U87
	any	brain	any	brain	any	brain			
RNF214	+	-	-	-	-	-	RNF214	+	+
BACE1	+	-	-	-	+	-	BACE1	+	+
CEP164	+	-	+	-	+	-	CEP164	+	+
DSCAML1	+	+	+	+	+	-	DSCAML1	+	-
FXYD6-FXYD2	-	-	-	-	-	-	FXYD6-FXYD2	-	-
TMPRSS13	+	-	+	-	-	-	TMPRSS13	-	-
IL10RA	+	-	-	-	-	-	IL10RA	-	-
TMPRSS4	+	+	-	-	-	-	TMPRSS4	-	-
SCN4B	+	-	-	-	-	-	SCN4B	-	-
SCN2B	+	-	-	-	+	-	SCN2B	-	-
AMICA1	+	+	-	-	-	-	AMICA1	-	+
MPZL3	+	+	-	-	-	-	MPZL3	+	-
CD3E	+	-	-	-	-	-	CD3E	-	-
CD3D	+	-	-	-	-	-	CD3D	-	-
CD3G	+	-	-	-	+	-	CD3G	-	-
UBE4A	+	-	-	-	+	-	UBE4A	+	+
ATP5L	+	-	-	-	-	-	ATP5L	+	+
KMT2A	+	+	+	-	+	-	KMT2A	+	+
TTC36	+	-	-	-	-	-	TTC36	+	-
TMEM25	+	-	+	-	+	-	TMEM25	+	-
IFT46	+	-	+	-	-	-	IFT46	+	+
ARCN1	+	-	-	-	+	+	ARCN1	+	+
PHLDB1	+	+	-	-	+	+	PHLDB1	+	+
TREH	+	-	-	-	-	-	TREH	-	-
DDX6	+	+	-	-	+	-	DDX6	+	+
CXCR5	+	-	-	-	+	-	CXCR5	-	-
BCL9L	+	+	+	-	+	-	BCL9L	+	+
UPK2	+	-	-	-	+	-	UPK2	-	-
FOXR1	+	-	-	-	-	-	FOXR1	-	-
CCDC84	+	-	-	-	-	-	CCDC84	+	+
RPL23AP64	-	-	-	-	-	-	RPL23AP64	+	+
RPS25	+	-	-	-	-	-	RPS25	+	+
TRAPPC4	+	-	-	-	-	-	TRAPPC4	+	+
SLC37A4	+	-	-	-	-	-	SLC37A4	+	+
HYOU1	+	-	-	-	+	+	HYOU1	+	+
VPS11	+	+	-	-	+	+	VPS11	+	+
HMBS	+	-	-	-	-	-	HMBS	+	+
H2AFX	+	-	-	-	-	-	H2AFX	+	+
DPAGT1	+	-	-	-	+	-	DPAGT1	+	+
C2CD2L	+	-	-	-	+	-	C2CD2L	+	+
HINFP	+	-	-	-	-	-	HINFP	+	+
ABCG4	+	+	-	-	+	-	ABCG4	+	-
NLRX1	+	-	+	-	+	-	NLRX1	+	+
PDZD3	+	+	-	-	+	-	PDZD3	-	-
CCDC153	+	+	+	-	-	-	CCDC153	-	-
CBL	+	+	-	-	-	-	CBL	+	+
MCAM	+	+	-	-	-	-	MCAM	+	+
RNF26	+	-	-	-	-	-	RNF26	+	+
MFRP	+	-	+	-	+	-	MFRP	-	-
THY1	+	-	-	-	+	+	THY1	+	+
PVRL1	+	-	+	-	+	-	PVRL1	+	+

Supplementary Figure 3: Somatic mutations and expression of 11q23.3 locus genes. Gene lists were entered into COSMIC database searches to obtain mutation data. Genes are listed in order from centromere to telomere. Gene promoter regions were visually inspected using available ENCODE data for promoter marks (H3K4me3) in NHA and RNA-seq in U87.

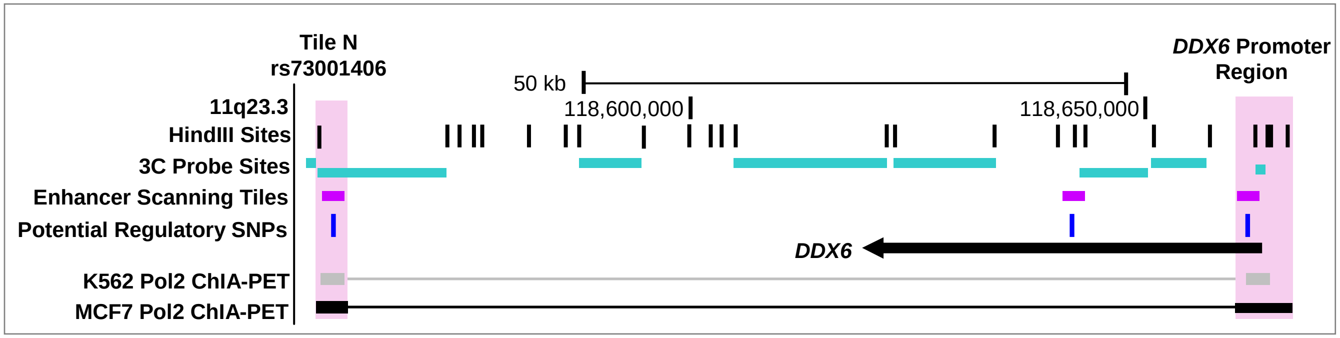

Supplementary Figure 4: ChIA-PET at the 11q23.3 locus. ENCODE was used to search for ChIA-PET data from available cell lines at the 11q23.3 locus. Shown are Pol2 ChIA-PET results for K562 and MCF7 cell lines at the 11q23.3 locus.

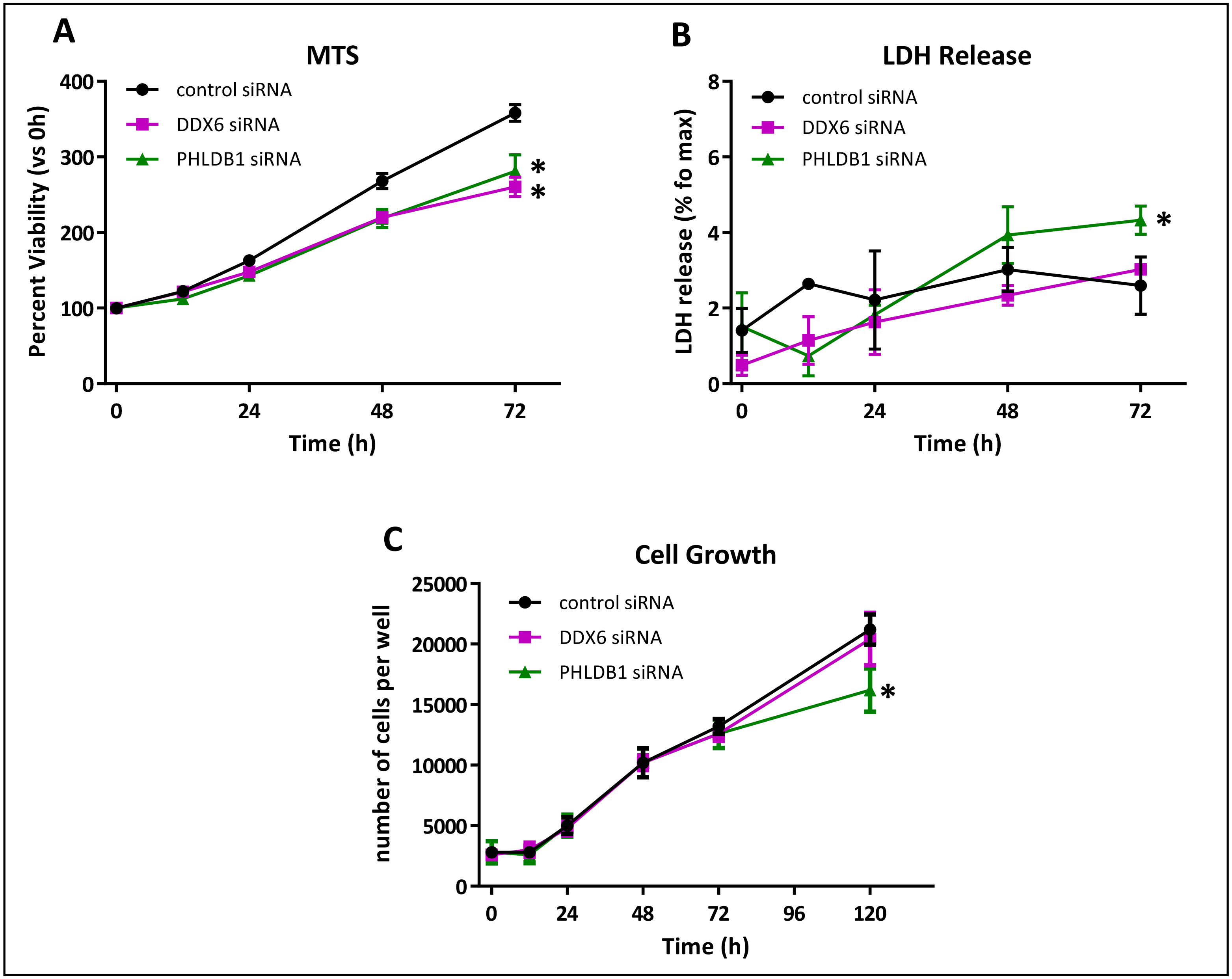

Supplementary Figure 5: Cell proliferation, cytotoxicity and viability assays. Cell proliferation (A) and LDH release (B) were measured up to 72h post-transfection. Cell viability (C) was measured by cell counts up to 120h post-transfection.

Supplementary Table 1: All candidate SNPs in the 11q23.3 locus

RSID	Rsquared to rs498872	Chromosome	Coordinate_HG19	Allele	MAF	Functional Evidence from Database Search?	Overlapping biofeatures in brain
rs1715445	0.222	chr11	118119187	C/T	0.195288		
rs12275284	0.227	chr11	118129727	A/T	0.318091		
rs576950	0.235	chr11	118391375	C/T	0.214257		
rs4938513	0.291	chr11	118419704	C/T	0.267372		
rs542266	0.229	chr11	118422240	A/C	0.292732		
rs633308	0.341	chr11	118459069	C/T	0.266973		
rs10736492	0.253	chr11	118459269	A/G	0.368411		
rs498872	1	chr11	118477367	C/T	0.245607	+	NHA_H3K27ac,_NHA_H3K4me1
rs7125115	0.268	chr11	118478330	A/G	0.440096	+	NHA_H3K27ac,_NHA_H3K4me1,_NHA_H3K4me3,_NHA_DNaseI_HS_peak
rs12225399	0.241	chr11	118480285	C/G	0.377995	+	NHA_H3K27ac,_NHA_H3K4me1,_NHA_H3K4me3,_NHA_DNaseI_HS_peak
rs10892246	0.262	chr11	118481431	A/G	0.361422	+	NHA_H3K27ac,_NHA_H3K4me1,_NHA_H3K4me3
rs112959066	0.386	chr11	118482565	A/T	0.172923	+	NHA_H3K27ac,_NHA_H3K4me1
rs45540840	0.507	chr11	118486110	A/G	0.242212	+	NHA_H3K27ac,_NHA_H3K4me1,_NHA_DNaseI_HS_peak
rs11216930	0.507	chr11	118488782	A/C	0.245208	+	NHA_H3K27ac,_NHA_H3K4me1,_NHA_H3K4me3
rs10892247	0.507	chr11	118490076	A/G	0.20008	+	NHA_H3K27ac,_NHA_H3K4me1,_NHA_H3K4me3
rs77683570	0.477	chr11	118495287	C/T	0.203874	+	NHA_H3K27ac,_NHA_H3K4me1
rs45565037	0.477	chr11	118496119	A/G	0.221046	+	NHA_H3K27ac,_NHA_H3K4me1
rs2236661	0.477	chr11	118499394	C/G	0.221645	+	NHA_H3K4me1
rs10892248	0.477	chr11	118501022	A/G	0.23103	+	NHA_H3K4me1
rs11216937	0.477	chr11	118507478	C/T	0.204872	+	
rs11216938	0.477	chr11	118514625	C/T	0.220447	+	
rs10790255	0.378	chr11	118515579	G/T	0.311302	+	
rs12362152	0.477	chr11	118516737	A/G	0.232228		
rs17748	0.477	chr11	118528424	C/T	0.203874	+	
rs7928371	0.477	chr11	118529127	A/G	0.220048	+	
rs2276065	0.477	chr11	118530611	C/T	0.216853	+	
rs10790256	0.477	chr11	118534082	C/T	0.197085	+	NHA_H3K27me3
rs12225548	0.477	chr11	118535840	C/G	0.203674	+	NHA_H3K27me3
rs10892251	0.413	chr11	118543563	C/T	0.207668	+	NHA_H3K27me3
rs2277296	0.383	chr11	118550522	C/T	0.226438	+	NHA_H3K27me3
rs2277297	0.383	chr11	118550524	C/T	0.226238	+	NHA_H3K27me3
rs10892252	0.383	chr11	118555692	A/G	0.207268	+	NHA_H3K27me3
rs11216943	0.383	chr11	118556398	A/G	0.216853	+	NHA_H3K27me3
rs73001406	0.383	chr11	118560857	A/G	0.229233	+	NHA_H3K27ac,_NHA_H3K4me1,_NHA_H3K4me3,_NHA_DNaseI_HS_peak
rs11216956	0.266	chr11	118575326	A/G	0.19988		
rs947931	0.209	chr11	118577614	A/C	0.0924521	+	NHA_H3K27me3
rs11216960	0.266	chr11	118577873	A/G	0.186102	+	NHA_H3K27me3
rs10892256	0.266	chr11	118578705	A/G	0.186502	+	NHA_H3K27me3
rs10892257	0.266	chr11	118579327	A/G	0.186901	+	NHA_H3K27me3
rs10790261	0.266	chr11	118579747	A/G	0.182109	+	NHA_H3K27me3
rs10892258	0.266	chr11	118579865	A/G	0.186701	+	NHA_H3K27me3
rs11216961	0.266	chr11	118580338	C/G	0.186901		
rs11216964	0.266	chr11	118581895	A/G	0.186701		
rs10892259	0.266	chr11	118583002	A/G	0.186302		
rs10892260	0.266	chr11	118583525	A/C	0.173323	+	
rs11216972	0.265	chr11	118585826	C/T	0.182308		
rs10892263	0.219	chr11	118587319	C/T	0.169529		
rs10892271	0.292	chr11	118598544	A/G	0.198682		
rs10892272	0.292	chr11	118602707	C/G	0.213858		
rs10892273	0.292	chr11	118602809	A/G	0.213858		
rs73001429	0.292	chr11	118603165	G/T	0.236422		
rs11216993	0.292	chr11	118603495	A/T	0.224641		
rs11216994	0.292	chr11	118603520	C/T	0.235623		
rs11216995	0.292	chr11	118603526	A/G	0.235623		
rs11216998	0.262	chr11	118604837	C/T	0.260583		
rs11216999	0.218	chr11	118604941	C/T	0.259784		
rs11217000	0.292	chr11	118604984	A/G	0.169129		
rs10892274	0.274	chr11	118605463	C/T	0.287141		
rs11217001	0.238	chr11	118607046	A/G	0.160543		
rs77209083	0.208	chr11	118607569	A/G	0.157548		
rs7945144	0.236	chr11	118608716	A/G	0.288339		
rs11826521	0.208	chr11	118610549	G/T	0.159145	+	NHA_H3K27me3
rs6589684	0.208	chr11	118610957	A/G	0.227636	+	NHA_H3K27me3
rs10892279	0.208	chr11	118611781	A/G	0.27476	+	
rs10892280	0.208	chr11	118611817	C/T	0.159145	+	
rs6589685	0.208	chr11	118612197	C/T	0.280351		
rs6589686	0.208	chr11	118612565	A/G	0.274161		
rs2155433	0.208	chr11	118612921	C/T	0.274361		
rs56758835	0.208	chr11	118615340	A/G	0.15615		
rs1048024	0.208	chr11	118618776	A/T	0.158546	+	
rs2077579	0.208	chr11	118619047	G/T	0.158746	+	
rs2004781	0.236	chr11	118619253	C/T	0.15595	+	
rs3889239	0.208	chr11	118619960	A/G	0.15595	+	
rs10892286	0.208	chr11	118642085	A/C	0.158546	+	NHA_H3K27ac,_NHA_H3K4me1
rs10892287	0.208	chr11	118642999	C/T	0.159545		
rs10892288	0.208	chr11	118644694	A/G	0.158347		
rs10892289	0.208	chr11	118646003	C/T	0.155751		
rs56043232	0.208	chr11	118648373	C/T	0.155751		
rs73003215	0.208	chr11	118649512	A/G	0.275759		
rs57494551	0.208	chr11	118661398	C/T	0.154752	+	NHA_H3K27ac,_NHA_H3K4me3
rs10892293	0.208	chr11	118665575	A/T	0.141973	+	
rs10892294	0.208	chr11	118667357	C/G	0.17492		
rs11217032	0.208	chr11	118669605	A/T	0.147963	+	NHA_H3K27me3
rs11217033	0.208	chr11	118673706	C/T	0.147764	+	NHA_H3K27me3
rs11217040	0.208	chr11	118680648	A/C	0.22504	+	NHA_H3K27me3
rs73005423	0.233	chr11	118681079	A/G	0.15595	+	NHA_H3K27me3
rs73005426	0.233	chr11	118681083	A/G	0.227835	+	NHA_H3K27me3
rs7942535	0.208	chr11	118681464	C/T	0.22524	+	NHA_H3K27me3
rs11217042	0.208	chr11	118682528	A/G	0.22524	+	NHA_H3K27me3
rs80065107	0.208	chr11	118682767	C/T		+	NHA_H3K27me3
rs76704408	0.208	chr11	118683327	G/T	0.234824	+	NHA_H3K27me3
rs17122453	0.208	chr11	118683564	A/G	0.22504	+	NHA_H3K27me3
rs715412	0.208	chr11	118684610	A/G	0.144569	+	NHA_H3K27me3
rs7123726	0.234	chr11	118694547	C/T	0.230232		
rs4936441	0.213	chr11	118725660	C/G	0.194888		
rs4938572	0.213	chr11	118740931	C/T	0.229633	+	

Supplementary Table 2: MATCH TFBS Analysis		
SNP	Factor Name	Allele Specificity
rs7125115	Elk-1	
	Oct-1	
	Ik-1	
	HNF-4	
	RFX1	
	c-Rel	
	HNF-3beta	Minor
	c-Myb	
	FOXD3	Minor
	v-Myb	
	Pax-4	
rs12225399	myogenin/NF-1	
	R	Major
	Pax-4	
	USF	
	N-Myc	
	c-Myc/Max	
rs45540840	GATA-3	
	Evi-1	
	Oct-1	Major
	GATA-1	Major
	HNF-4	Major
	GATA-2	Major
	Pax-4	
	Lmo2_complex	Major
	GATA-X	Major
	Hand1/E47	
	ARP-1	Minor
rs11216930	Oct-1	
	HNF-4	
	Oct-1	
rs10892247	Hand1/E47	
	Hand1/E47	
	HNF-4	
	Hand1/E47	
	GATA-3	Major
	v-Maf	
	CP2	
	GATA-X	
	c-Rel	
	CP2	
	GATA-2	
	c-Ets-1(p54)	Minor
rs2236661	Hand1/E47	
	myogenin/NF-1	
	Staf	
	COMP1	
	CDP CR1	
	USF	Major
	Oct-1	
rs10892248	Pax-4	
	AREB6	
	AP-1	
	GATA-X	
	GATA-1	
	Lmo2	
rs73001406	c-Rel	
	COMP1	
	Elk-1	
	HNF-1	
	Hand1/E47	
	Pax-6	Major
	Pax-4	
	Ik-1	
	Ik-3	
	Oct-1	
	N-Myc	Major
	USF	Major
	AP-1	
rs10790261	COMP1	
	Hand1/E47	Major
	Olf-1	Major
	SOX-9	
	NF-Y	
	c-Ets-1(p54)	Minor
rs10892258	Pax-4	
	CDP CR1	
	Evi-1	
	Pax-6	Minor
	AP-1	
	HNF-4	
	ER	
	Oct-1	
	FOXD3	
	HNF-1	
	HLF	
rs57494551	myogenin/NF-1	Major
	COMP1	Major
	Evi-1	
	Oct-1	
	Elk-1	

Supplementary Table 3: Enhancer scanning tile primers

Amplicon name	FW Primer	REV Primer	Amplicon Size	SNPs covered	BAC
11A	CTAGAGTCTCGCTCCTCAAA	CGGGAGAGTCTGGATAGG	1835	rs498872 rs7125115	RP11-45N4
11B	CACAAGGGCGTCTTCTTT	GAGGTTTCCGCAAGGTAG	2347	rs7125115 rs12225399	RP11-45N4
11C	ACACACAGCCCCAGTTATAC	AGCTGTGGTGTGCTCATT	2272	rs12225399 rs10892246	RP11-45N4
11D	TTGGATCAGGTTACTGTGC	GGGTTCTTAATTGGCTAGGT	1868	rs112959066	RP11-45N4
11E	GTGACTGGTTTAGGGTGGTA	TTTGGGTTATATTCGTGACC	2223	rs45540840	RP11-45N4
11F	CAAGTAGCTGGGATTACAGG	GAGGAAAAAGGAAGAACCAT	2137	rs11216930 rs10892247	RP11-45N4
11G	GTGTGTGAGAGACGGAGTTT	CAAACAGCTAAGGGAGTGAC	2102	rs77683570 rs45565037	RP11-45N4
11H	CAGGGAAGTGGCAGAGAGTC	CCCACCCAGATACAGTCACC	2116	rs2236661 rs10892248	RP11-45N4
11I	GCGCTCACCAGTAGTAGAAC	GGGCCATTGTACAGAGAAT	1894	rs10790256	RP11-45N4
11J	TTGGGCAGTAAAGTACCTGT	CCACTGTATTGGGTAGGAAA	1952	rs12225548	RP11-45N4
11K	AAGCATGCCTACAGGATCTA	TGCAAATGCTCAAAATACTG	1768	rs10892251	RP11-45N4
11L	CTCTAACACACCCTCTGCTC	TGAAACCCCGTCTCTACTAA	2147	rs2277296 rs2277297	RP11-45N4
11M	AATGCCCTGTTTTATTTTGA	CCTGTAGTCCCAGCTATTTG	2160	rs10892252 rs11216943	RP11-45N4
11N	CTAAGACCCGGTGACAAAT	GTAGGACTAGGGACGGGATA	1816	rs73001406	RP11-45N4
11O	AGATGTCATTGTCCCTTGAG	AAAGTTTGCTTCCCATTGTA	2437	rs947931 rs11216960 rs10892256 rs10892257	RP11-45N4
11P	CATCTCTGACCAGGAACATT	GCATCTGTAGTCCCAGCTAC	1843	rs10790261 rs10892258	RP11-45N4
11Q	CATGGTGAAACCTCATCTCT	TCATGACCACACAAAGAAAA	2220	rs11826521 rs6589684	RP11-45N4
11R	TGACATTTGTCCCTTCTATG	TTTATCCAGTCATCAGTTGG	1901	rs10892286	RP11-45N4
11S	ACCTCTAAAGACCGAACGA	CAAGATACGCCCTCACAG	2468	rs57494551	CTD-2333F20
11T	AGGCTGCAGTGAGTTATGAT	CTGAGGATAAACAGGAGCAC	1836	rs11217032	CTD-2333F20
11U	TTTCAAATGCTGTTGTCAAG	AGCAAGATCCTGTCTCAGAA	2173	rs76704408 rs17122453 rs715412	CTD-2333F20
NEGATIVE	TCTACCTGGGACTCATTGAC	CCCTTGGCTTCACTTCTAA	1784	n/a	RP11-45N4

attB sites are added to each FW and REV primer as follows:

attB FW
5' GGGGACAAGTTTGTACAAAAAAGCAGGCTACGCGT

attB REV
5’ GGGGACCACTTTGTACAAGAAAGCTGGGCTCGAG

Supplementary Table 4: EMSA probes

rs498872	major FW	5'-	CTTGCAAAGCCAGCTCTTGGCCCAGGAGACTGGCTGGGGAG
rs498872	major REV	5'-	CTCCCCAGCCAGTCTCCTGGGCCAAGAGCTGGCTTTGCAAG
rs498872	minor FW	5'-	CTTGCAAAGCCAGCTCTTGGTCCAGGAGACTGGCTGGGGAG
rs498872	minor REV	5'-	CTCCCCAGCCAGTCTCCTGGACCAAGAGCTGGCTTTGCAAG
rs7125115	major FW	5'-	AGCTCCCGGGGAAAAGCAACGGTGTCCTCCTAAGCCTGAGG
rs7125115	major REV	5'-	CCTCAGGCTTAGGAGGACACCGTTGCTTTTCCCCGGGAGCT
rs7125115	minor FW	5'-	AGCTCCCGGGGAAAAGCAACAGTGTCCTCCTAAGCCTGAGG
rs7125115	minor REV	5'-	CCTCAGGCTTAGGAGGACACTGTTGCTTTTCCCCGGGAGCT
rs12225399	major FW	5'-	CAGCTCACACCGCAGCCCCAGCTCACACACGCGGCCCCTCA
rs12225399	major REV	5'-	TGAGGGGCCGCGTGTGTGAGCTGGGGCTGCGGTGTGAGCTG
rs12225399	minor FW	5'-	CAGCTCACACCGCAGCCCCACCTCACACACGCGGCCCCTCA
rs12225399	minor REV	5'-	TGAGGGGCCGCGTGTGTGAGGTGGGGCTGCGGTGTGAGCTG
rs45540840	major FW	5'-	AGGGCAAGATCTCCCAAGATGAGGCCCTGGCACAGCCCCCT
rs45540840	major REV	5'-	AGGGGGCTGTGCCAGGGCCTCATCTTGGGAGATCTTGCCCT
rs45540840	minor FW	5'-	AGGGCAAGATCTCCCAAGATAAGGCCCTGGCACAGCCCCCT
rs45540840	minor REV	5'-	AGGGGGCTGTGCCAGGGCCTTATCTTGGGAGATCTTGCCCT
rs11216930	major FW	5'-	TAGCTCCCTGAGAAGGGGAGAGCCTAGGGCTAAGATGGAAT
rs11216930	major REV	5'-	ATTCCATCTTAGCCCTAGGCTCTCCCCTTCTCAGGGAGCTA
rs11216930	minor FW	5'-	TAGCTCCCTGAGAAGGGGAGCGCCTAGGGCTAAGATGGAAT
rs11216930	minor REV	5'-	ATTCCATCTTAGCCCTAGGCGCTCCCCTTCTCAGGGAGCTA
rs10892247	major FW	5'-	AGGAGTGTGGCTGGGGAGCCGGATCTCTGGGTTATCCAGC
rs10892247	major REV	5'-	GCTGGATAACCCAGAGATCCGGCTCCCCAGCCACACTCCT
rs10892247	minor FW	5'-	AGGAGTGTGGCTGGGGAGCCAGATCTCTGGGTTATCCAGC
rs10892247	minor REV	5'-	GCTGGATAACCCAGAGATCTGGCTCCCCAGCCACACTCCT
rs2236661	major FW	5'-	CAGAGACTCTGCCATGGACACTGGCAAGCCTCCCTGGCACC
rs2236661	major REV	5'-	GGTGCCAGGGAGGCTTGCCAGTGTCCATGGCAGAGTCTCTG
rs2236661	minor FW	5'-	CAGAGACTCTGCCATGGACAGTGGCAAGCCTCCCTGGCACC
rs2236661	minor REV	5'-	GGTGCCAGGGAGGCTTGCCACTGTCCATGGCAGAGTCTCTG
rs10892248	major FW	5'-	CCTTTACCTCCTAGGCCCCTGAGAGGTGACTGTATCTGGGT
rs10892248	major REV	5'-	ACCCAGATACAGTCACCTCTCAGGGGCCTAGGAGGTAAAGG
rs10892248	minor FW	5'-	CCTTTACCTCCTAGGCCCCTAAGAGGTGACTGTATCTGGGT
rs10892248	minor REV	5'-	ACCCAGATACAGTCACCTCTTAGGGGCCTAGGAGGTAAAGG
rs73001406	major FW	5'-	AGAAGGTTCCAGACATTCCCGTGACTGTGGGTGGGGCTGGA
rs73001406	major REV	5'-	TCCAGCCCCACCCACAGTCACGGGAATGTCTGGAACCTTCT
rs73001406	minor FW	5'-	AGAAGGTTCCAGACATTCCCATGACTGTGGGTGGGGCTGGA
rs73001406	minor REV	5'-	TCCAGCCCCACCCACAGTCATGGGAATGTCTGGAACCTTCT
rs10790261	major FW	5'-	AATGTACAAGATGTGTCTCCAGTCCCAATGGTAGACATCCT
rs10790261	major REV	5'-	AGGATGTCTACCATTGGGACTGGAGACACATCTTGTACATT
rs10790261	minor FW	5'-	AATGTACAAGATGTGTCTCCGGTCCCAATGGTAGACATCCT
rs10790261	minor REV	5'-	AGGATGTCTACCATTGGGACCGGAGACACATCTTGTACATT
rs10892258	major FW	5'-	CTGAGGATCAGTCAAGCTCAGAGCAAGATTGAATAACCCCT
rs10892258	major REV	5'-	AGGGGTTATTCAATCTTGCTCTGAGCTTGACTGATCCTCAG
rs10892258	minor FW	5'-	CTGAGGATCAGTCAAGCTCAAAGCAAGATTGAATAACCCCT
rs10892258	minor REV	5'-	AGGGGTTATTCAATCTTGCTTTGAGCTTGACTGATCCTCAG
rs57494551	major FW	5'-	ACCCTGCTGCGCACCACATTCCTATATTCGCCTCCTCTCAG
rs57494551	major REV	5'-	CTGAGAGGAGGCGAATATAGGAATGTGGTGCGCAGCAGGGT
rs57494551	minor FW	5'-	ACCCTGCTGCGCACCACATTTCTATATTCGCCTCCTCTCAG
rs57494551	minor REV	5'-	CTGAGAGGAGGCGAATATAGAAATGTGGTGCGCAGCAGGGT

Supplementary Table 5: 3C primers

Hg19 chr11 coordinate	Primer name	Sequence
118559230	Bait	5'-CGAACCACCAGCGGCAATC
118559075	Adjacent	5'-CTCGTGGTTTCGCTGGCTTC
118587850	1	5'-GGCCCCACCCCTATCTCTCT
118605130	2	5'-AGACAGCCCCGTTCCAACAT
118622535	3	5'-TTCTGGGGAAAGGGGACAAGG
118633460	4	5'-AGTCCTGGGATCTAGCCATGT
118651050	5	5'-GAGCCGAGATCACGCCACT
118663145	6	5'-GAAAGTAGGGGCGGAGGAGG
118546270	7	5'-GCACCCCTTCATTTGGCTGA
118504220	8	5'-GAGGGCAGGCAGGGGAATAG
118489020	9	5'-GGGGAGGACTGGGGAGAAGA
118475695	10	5'-GCCCCTGTCCTGGTTTGGTA
	control primer 1 (FW)	5'-GTTCATCTTGCTGCCAGAAATGCCGAGCCTG
	control primer 2 (REV)	5'-ATCCCAGCTGTCTGTAGCTTTAGAAAGTGGG

Supplementary Table 6: 3C results

Raw Data: DNA Quantification

Adjacent	1	2	3	4	5	6	7	8	9	10
0.01857	2.55E-06	8.25E-05	0.001712	0.010903	0.235545	0.034679	1.12E-05	0.001401	3.28E-04	0
0.014368	1.82E-08	1.57E-04	0.007697	4.09E-05	0.481944	0.034549	1.28E-06	4.52E-04	5.13E-04	0
0.020004	1.02E-08	2.56E-05	0.003734	1.71E-05	0.347292	0.02223	6.65E-06	7.21E-04	0.001735	3.32E-07
0.014552	2.46E-08	1.63E-04	0.004199	1.50E-05	0.364878	0.024361	1.40E-05	0.001511	3.86E-04	0
0.022799	2.80E-08	2.49E-04	0.005069	4.51E-05	1.004076	0.100759	6.34E-05	0.001047	4.08E-04	3.55E-07
0.019766	2.28E-08	1.68E-04	0.005562	1.18E-04	0.805752	0.089343	4.45E-05	6.69E-04	8.92E-04	2.56E-07

Normalized against adjacent

Adjacent	1	2	3	4	5	6	7	8	9	10
1.012367	0.000139	0.004498	0.093348	0.594392	12.84099	1.890556	0.000608	0.076387	0.017905	0
0.783308	9.92E-07	0.008546	0.419619	0.002229	26.27371	1.883503	7E-05	0.024621	0.027948	0
1.090555	5.56E-07	0.001394	0.203582	0.00093	18.93302	1.21188	0.000363	0.039317	0.094558	1.81E-05
0.793317	1.34E-06	0.008885	0.228922	0.000818	19.8917	1.328072	0.000761	0.082376	0.021026	0
1.242914	1.53E-06	0.01358	0.276326	0.00246	54.73827	5.492986	0.003454	0.057097	0.02226	1.93E-05
1.077539	1.24E-06	0.009167	0.303221	0.006434	43.92647	4.870604	0.002427	0.036473	0.048639	1.39E-05
